# Notes and Notices

**Published:** 1898-10

**Authors:** 


### NOTES AND NOTICES.

The New York Post-Graduate Medical School and Hospital, University of the State of New York, has just issued the seventeenth annual announcement for 1898 and 1899. It shows that five hundred and twenty-three practitioners of medicine have attended its courses during the past year. They came from the various States of the Union and the Dominion of Canada. There were ten physicians from foreign countries, two of these being from India and one from Japan. Only ninety-six were from the State of New York.

Acetate of Thallium.—At a meeting of the Paris Academy of Medicine, Dr. Huchard read a paper on the use of "Acetate of Thallium" as a medicament against profuse perspiration in certain cases of serious illness, but produced photographs to prove that it causes the hair to fall off with great rapidity. Patients had become quite bald in several days. A new remedy for this troublesome affection which is by no means well provided for.—*Homœopathic World*, June 4th, 1898.

See a lot of Portuguese men on another page treading grapes with their feet for making wine. Read about it, also about Speer's improved method of mashing grapes and making wine.

Eclectic College Confederation.—At the annual meeting of the National Eclectic Medical Association, held at Niagara Falls, N. Y., June 19th, 20th and 21st, 1894, the following resolution, recommended by the Committee on Medical Colleges, was adopted unanimously:

"*Resolved*, That the Committee on Medical Colleges recommend the organization of an Eclectic Medical College Association, composed of two delegates from each college recognized by the National Association; and that the Committee on Medical Colleges be given power to sanction any action taken by said organization." H. Wolgemuth, M. D., Chairman; J. K. Scudder, M. D.; E. Younkin, M. D.; V. A. Baker, M. D.; H. H. Green, M. D., Committee.

Following the adoption of the above, the "National Confederation of Eclectic Medical Colleges" has organized, and a constitution adopted, of which Article II is herewith appended:

"The objects of this Confederation shall be to maintain organized co-operation between the Eclectic Medical Colleges recognized by the National Eclectic Medical Association; for the purpose of promoting the mutual interests of said colleges, establishing uniform minimum requirements and curriculum, and furthering the cause of higher medical education."

For the Weak and Aged.—The best thing for weakly persons and invalids is Speer's Port Grape Wine. His Burgundy and Claret Wines are used at dinner by the best society people in New York and Washington.

Pulte Medical College.—The principal features of the college are briefly these: Six full months of instruction, four separate years. On this course one year is allowed to graduates in dentistry, pharmacy, and veterinary medicine, and to students holding Bachelor's degrees from recognized colleges. Students from other medical colleges are admitted to that year in this college which their credentials may entitle. The curriculum is carefully graded, and there is no unnecessary repetition of work. Thorough quizzes and reviews are frequent. In the first two years the course is largely laboratory work and recitations. In the last two years it is largely clinical. Clinical advantages are unsurpassed. The Central Deaconess Hospital staff are also connected with Pulte Medical College; the Cincinnati Hospital, the Home for the Friendless and Foundlings, and the Cincinnati Homœopathic Free Dispensary furnish an abundance of first-class clinical instruction.

See a lot of Portuguese men on another page treading grapes with their feet for making wine. Read about it, also about Speer's improved method of mashing grapes and making wine.

John Storer, M. D., has removed from Jamaica Plain, Mass., to Chicago. His address is 809 Columbus Mem. Blg., corner State and Washington Streets, Chicago. Hours: 10 A. M. to 1 P. M. Telephone, Main 32. Residence, 108 S. Oak Park Ave., Oak Park, Ill. Hours: 8–9 A. M., 5–7 P. M.; Sundays, 12–1. Telephone, Oak Park 1272.

Noah After the Flood planted the first fruit, the grape, the most healthy of al the products of the earth.

Speer, the oldest wine grower in the U. S., has vineyards of the Portugal Grape, from which his wines are made and fully matured by great age, and are valuable.

